# When does the “third fluid space” open?

**DOI:** 10.1007/s00424-025-03135-y

**Published:** 2025-12-17

**Authors:** Robert G. Hahn

**Affiliations:** Karolinska Institutet at Danderyds Hospital (KIDS), Stockholm, 171 77 Sweden

**Keywords:** Crystalloid fluid, pharmacokinetics, Interstitial fluid, Hemodilution, Water–electrolyte balance, physiology

## Abstract

Recent kinetic studies show that the interstitial space contains two functional fluid compartments. This study explores the prerequisites for accumulation of infused crystalloid fluid in the remote slow-exchange space (“third fluid space¨, *V*_t2_). Volume kinetic analysis based on log likelihood mathematics was applied to retrospective data from 132 intravenous infusions in 85 healthy volunteers who received 0.6–2.5 L of crystalloid fluid over 30 min. Frequent measurements of the blood hemoglobin concentration during and after these infusions, together with the measured urine output, served as calculation inputs. Three substudies were set up to illustrate key issues regarding *V*_t2_ filling. In the first of them, infusions were preceded by blood withdrawal, which is known to decrease the interstitial pressure (P_if_). Blood withdrawal resulted in smaller volumes entering *V*_t2_, confirming that *V*_t2_ filling is dependent on P_if_. In the second substudy, modeled and measured urine outputs were compared after varying the inclusion of *V*_t2_ in kinetic analyses in which the infused volume was gradually increased. Consideration of *V*_t2_ was deemed appropriate when > 1.2 L of fluid was administered. In the third substudy, assessment of *V*_t2_ filling during 7 time periods just before and after the 30 min infusions confirmed that uptake of fluid to *V*_t2_ was initiated between 30 and 35 min. In conclusion, accumulation of fluid in *V*_t2_ is dependent on P_if_ and occurs following infusion of > 1.2 L of crystalloid fluid. Uptake is not gradual but is initiated at a specific point in time.

**Supplementary Information:**

The online version contains supplementary material available at 10.1007/s00424-025-03135-y.

## Introduction

The concept of a “third fluid space,” introduced by Shires et al. in the 1960s, denotes the existence of a second interstitial body compartment in which fluid accumulates during trauma and major surgery [1, 2]. This compartment was long considered a technical artifact due to flawed technology [3, 4]; however, recent volume kinetic studies demonstrate that the interstitial space indeed consists of two fluid compartments that are connected in series with the plasma — one exchanges fluid with the plasma at a high rate (*V*_t1_) and the other exchanges more slowly (*V*_t2_) [5–7]. The slow-exchange compartment seems to open for fluid accumulation when *V*_t1_ has increased by 600–800 mL and is probably due to the increase in the interstitial pressure (P_if_) caused by a fast intravenous infusion of crystalloid fluid. The value of P_if_ is normally slightly subatmospheric (i.e., −2 to −4 mmHg), because interactions between fibroblasts and the collagen matrix actively compress the interstitium to achieve a minimal volume that offers high resistance to fluid flow [8]. Experiments by Guyton et al. reported a dramatic decrease in the interstitial resistance to volume expansion when P_if_ exceeds zero [9–11], and the current belief is that the same event marks the opening of *V*_t2_.

Fluid accumulation in *V*_t2_ seems to operate as an overflow reservoir, and it greatly extends the half-life of the infused fluid. The clinician might be encouraged to intensify the fluid therapy because *V*_t2_ is kinetically well separated from the plasma and unlikely to provide hemodynamic support [7]. However, observational surgery and intensive care studies show that the subsequent fluid overload increases morbidity and mortality [12–16], although the pathophysiological mechanisms have often been unclear. In animal studies, the infusion of large amounts of isotonic saline and irrigating fluid damages the cytoarchitecture, creating scattered islands of free fluid (lacunae) in the skin and in the heart that are associated with acute mortality [17–19]. To what degree this tissue disruption is due to the opening of *V*_t2_ is unknown.

The aim of the present study was to explore the physiological prerequisites for the opening of *V*_t2_. The chosen method of study is volume kinetics, as this is a minimally invasive whole-body approach that allows calculations of how infused fluid distributes in living humans [20]. Three approaches were used. First, the changes in the “trigger” for opening of *V*_t2_ were explored when the pressure in *V*_t1_ is manipulated by withdrawing blood [21]. Second, differences between kinetic models were used to estimate what infusion volume is required before *V*_t2_ begins to accumulate fluid. Third, covariance analysis in mixed models mathematics was used to study whether opening of *V*_t2_ occurs gradually or suddenly. The hypothesis was that *V*_t2_ opens suddenly when a sufficient “threshold” filling of *V*_t1_ has been reached.

## Methods

The data were derived from a database of intravenous infusion experiments performed in volunteers during the past 25 years. The author planned and supervised all the studies, and all were performed using similar protocols. No subjects had restricted kidney function or severe cardiovascular disease. No subject received adrenergic drugs. Publications in which data have appeared previously, and their associated ethics approvals, are listed in Table S1 of **Supplementary File 1**. Informed consent was obtained from all participants.

### Study 1

Ten healthy men with a mean age of 28 years (range 23–33 years) and a mean body weight of 76 kg (65–85 kg) each underwent three fluid infusion experiments in random order, performed at least one week apart [22, 23]. All infusions consisted of 25 mL/kg of Ringer’s solution that was administered over 30 min. One experiment was performed in the normovolemic state and the others immediately after withdrawing either 450 mL or 900 mL of blood. The blood was returned upon completion of the experiment.

### Study 2

Ringer’s acetate was given in volumes between 0.6 and 2.5 L over 30 min to 101 healthy volunteers (24% women) aged between 19 and 45 (mean, 30) years and having a mean body weight of 76 kg (range 50–100). Venous blood was collected using the same protocol described in Study 1. The subjects voided in the recumbent position whenever needed, and the total voided volume was summarized at the end of the study.

Analysis was first performed for screening purposes and was then based on all data in a single run. Thereafter, analyses were performed separately in four volume ranges of infused fluid (0.6–1.0, 1.0–1.5, 1.5–2.0, and 2.5–2.5 L). The *post hoc* kinetic outputs from these analyses were then further divided into a total of 7 ranges. The predicted and measured urine outputs were compared at the end of the experiments, which typically concluded 180–240 min after the infusions were initiated.

### Study 3

Crystalloid fluid 25 mL/kg was given over 30 min to 102 volunteers (Ringer’s = 92, isotonic saline = 10) in volumes exceeding the minimum amount that opened *V*_t2_ according to Study 2. The volunteers were the same as those in Study 2, except that the 15 experiments with the lowest infused volumes were replaced with experiments in which larger volumes were administered. The participants had a mean age of 30 years (range 19–45) and a body weight of 78 (range 50–101) kg. The mathematical procedures were the same as those described in the first two sub-studies.

### Measurements

Venous blood was collected every 5 min for 60 min just before the fluid infusions were initiated and then every 10 min for up to 180 min after the infusion started (for a total of 24 time points). The blood was subsequently analyzed using a Technicon H2 analyzer (Bayer, Tarrytown, NY). Duplicate blood samples drawn at baseline in all experiments ensured coefficients of variation (CV) of 1.0% for this analysis. The subjects voided in the recumbent position, and the total voided volume was measured at the end of the study. The arterial pressure was measured noninvasively at 15–30 min intervals.

### Kinetic analysis

A three-volume kinetic model with five rate constants (*k*_12_, *k*_21_, *k*_23_, *k*_32_, and *k*_10_) and one scaling factor between dilution and central volume (*V*_c_) was fitted to the dependent variables (i.e., the frequently measured plasma dilution and total urinary excretion) in each sub-study [7].

**Fig. 1:**
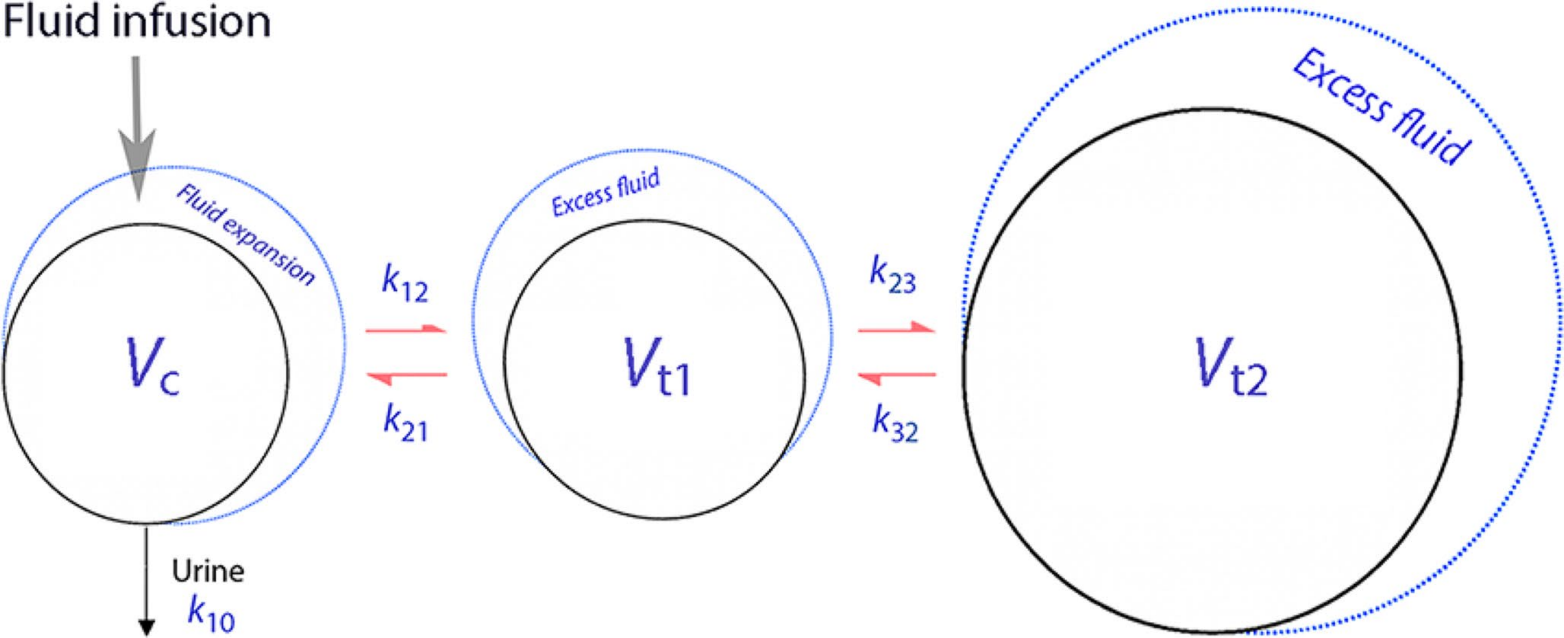
Schematic drawing of the kinetic model used to analyze the data

In this model, fluid is infused at the rate *R*_o_ into the plasma (*V*_c_), from where it is then distributed to (*k*_12_) and redistributed from (*k*_21_) the fast-exchange extravascular space (*V*_t1_). Fluid can also be translocated from *V*_t1_ to the slow-exchange interstitial fluid space (the “third fluid space”, *V*_t2_). Elimination occurs from *V*_c_ by urinary excretion (*k*_10_). All flow rates are proportional to the volume expansion of the respective body fluid space by the rate constant that signifies the flow (Fig. 1). The differential equations for the model are as follows:

$$\begin{array}{c}dv_c/dt=R_o-k_{10}\left(v_c-V_c\right)-k_{12}\left(v_c-V_c\right)+k_{21}\left(v_{t1}-V_{t1}\right)\\dv_{t1}/dt=k_{12}\left(v_c-V_c\right)-k_{21}\left(v_{t1}-V_{t1}\right)-k_{23}\left(v_{t1}-V_{t1}\right)+k_{32}\left(v_{t2}-V_{t2}\right)\\dv_{t2}/dt=k_{23}\left(v_{t1}-V_{t1}\right)-k_{32}\left(v_{t2}-V_{t2}\right)\\dU/dt=k_{10}\left(v_c-V_c\right)\end{array}$$

Baseline volumes are given in capital letters (*V*_c_ and *V*_t_), expanded volumes in lower-case letters (*v*_c_ and *v*_t_), and U is the measured urinary output. Hence, the volume expansion of the central fluid space is given by (*v*_c_ – *V*_c_).

The Hb-derived fractional plasma dilution used to indicate the volume expansion of *V*_c_ resulting from the infusion was given by the following (where Hct = baseline hematocrit):

$$\left(v_c-V_c\right)/V_c=\left[\left(Hb/hb\right)-1)\right]/\left(1-Hct\right)$$

The *exponential covariate model* was chosen to identify kinetic differences between the three series of experiments in Study 1 and between different time points for the opening of *V*_t2_ in Study 3. For example, the rate parameter *k*_23_ had a group value of 0.030 min^−1^ in the normovolemic state. Withdrawal of 450 mL blood was associated with a covariate effect of −0.22 that changed the value of *k*_23_ in this hemorrhaged group as follows:

$$k_{23}=0.030\left(e^{-0.22}\right)=0.024$$

where e = 2.718. The value of *k*_23_ is then 80% of that obtained in the normovolemic state.

The *power model* is appropriate for positive continuous data. Here, it was used to correct the size of *V*_c_ for variations in body weight. The covariance value is the exponent of the ratio between the individual body weight and the mean body weight for the group.

The kinetic model was simultaneously fitted to all measurements of plasma dilution and urinary excretion (dependent variables) in each substudy separately using the Phoenix software for nonlinear mixed effects (NLME), version 1.3 (Pharsight, St. Louis, MO) with the First-Order Conditional Estimation Extended Least Squares (FOCE ELS) as search routine. The sandwich method was employed as the variance estimator because it is robust for covariance misspecification. The goodness-of-fit and performance of the model were evaluated using predictive checks, residual plots, and conditional weighted residuals (CWRES) [24].

#### Statistics

Demographic data were reported as the mean and standard deviation (SD) and occasionally as the mean and the interquartile range (IQR). Kinetic parameters were reported as the best estimate (95% confidence interval) according to the output from the Phoenix program. The Akaike information criterion was used to decide whether the output should be presented according to a two- or three-volume model [25]. The criterion for accepting a covariate was that its inclusion should reduce the − 2 LL (log likelihood) for the model by > 3.84 points (*P* < 0.05) or > 6.6 points (*P* < 0.01). The significance levels for inclusion of the covariates were taken from the Phoenix program. A value of *P* < 0.05 was considered statistically significant.

## Results

### Study 1

A total of 1,855 ± 157 mL (mean ± SD) of Ringer’s was infused, and 683 measurements of plasma dilution were made. Urine output was measured at the end of each 180-min experiment.

All three series of experiments in Study 1 were analyzed in a single run using the model shown in Fig. 1. The three-volume model was statistically justified, given the Akaike criterion of −2361 (two-volume model − 2341). The goodness-of-fit and performance measures are reported in Fig. S1 and the kinetic output in Table S2**.** The original data are given in **Supplementary File 2.**

**Fig. 2:**
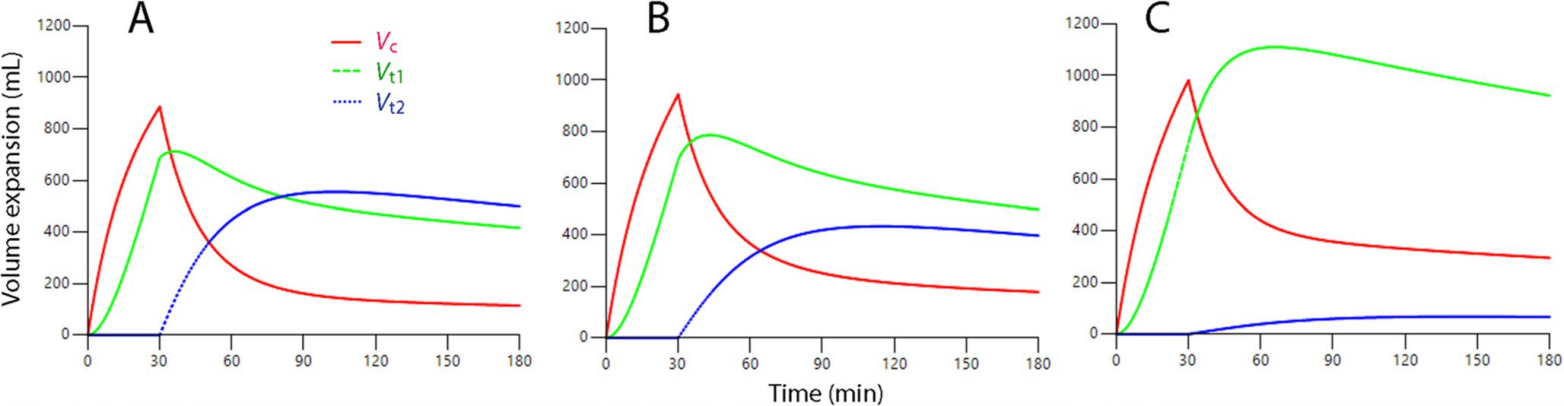
Simulated distribution of an infusion of 1,855 mL of Ringer’s acetate over 30 min in 10 healthy male volunteers with and without manipulation of the blood volume **(A)** No manipulation **(B)** After withdrawal of 450 mL of blood (**C**) After withdrawal of 900 mL of blood

The influence of hemorrhage on the kinetic parameters was quantified by covariance analysis. The results show that hemorrhage decreased *V*_c_, *k*_10_, and *k*_23_ in a graded fashion. The distribution of the infused fluid in the three experiments is shown in Fig. 2. Greater hemorrhage was associated with allocation of more of the infused fluid to *V*_t1_ while progressively less volume entered *V*_t2_. Only small amounts of fluid entered *V*_t2_ when the pre-infusion hemorrhage volume was 900 mL.

**Fig. 3:**
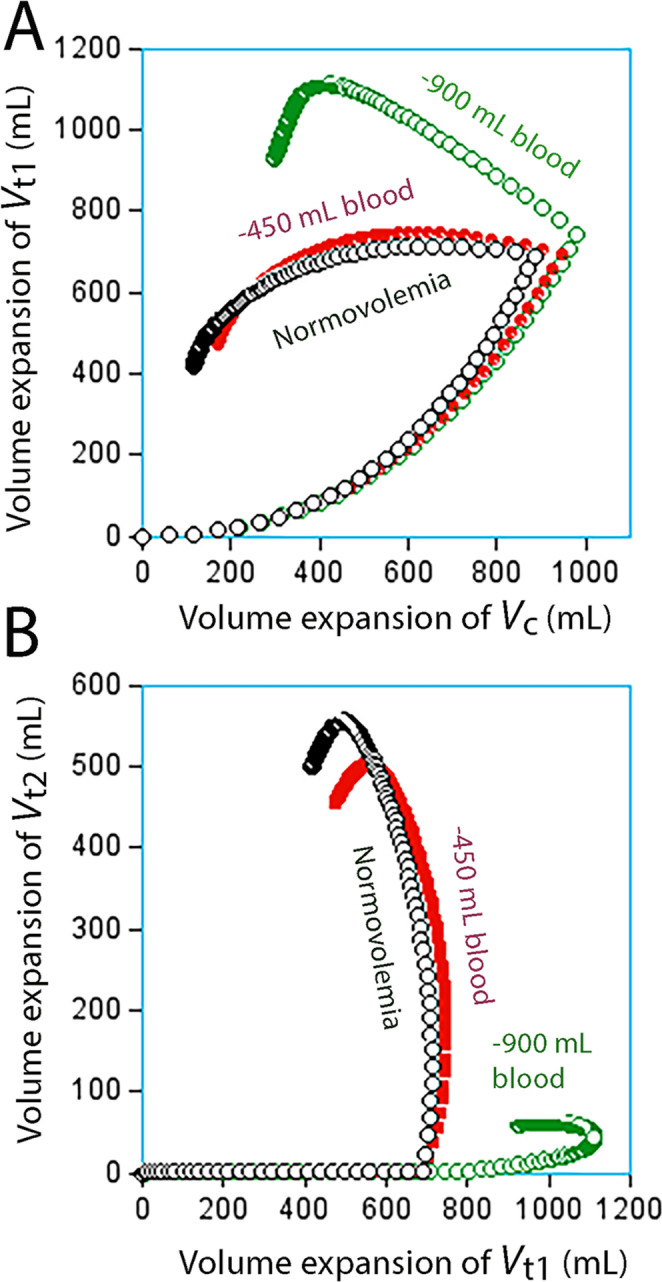
Simulated volume expansion of *V*_c_, *V*_t1_ and *V*_t2_ every minute for 3 h based on the “best” estimates of the kinetic parameters, shown in Table S2, obtained during the hemorrhage experiments. **(A)** Opening of *V*_t2_ occurred when *V*_t2_ had been expanded by 850–900 mL, and this event prevented further expansion of *V*_t1_ in normovolemic and moderately hemorrhaged volunteers **(B)** Opening of *V*_t2_ initiated a continuous expansion of this compartment, which continued almost to the end of the 3 h experimental period

Figure 3A illustrates that opening of *V*_t2_ effectively prevented further expansion of *V*_t1_, while Fig. 3B shows that further volume expansion only occurred in *V*_t2_. Opening of *V*_t2_ occurred when *V*_t1_ had expanded by 700 mL, but the threshold seemed to be slightly higher when the pre-infusion hemorrhage volume was 900 mL. However, the data material was underpowered to identify changes in the point of time when *V*_t2_ opened depending on the hemorrhage volume. A strong inhibition of *k*_23_ was evident as long as the 30 min infusions were ongoing, but explorative analyses suggested that this inhibition could end at any time between 25 and 45 min – prolonging or shortening the period for the covariance within this range resulted in very little change in its value. Study 3 is a focused analysis of this specific issue.

### Study 2

The infused volume of Ringer’s ranged from 625 mL to 2,443 mL (mean, 1,643) and 2,792 measurements of plasma dilution were made.

**Fig. 4:**
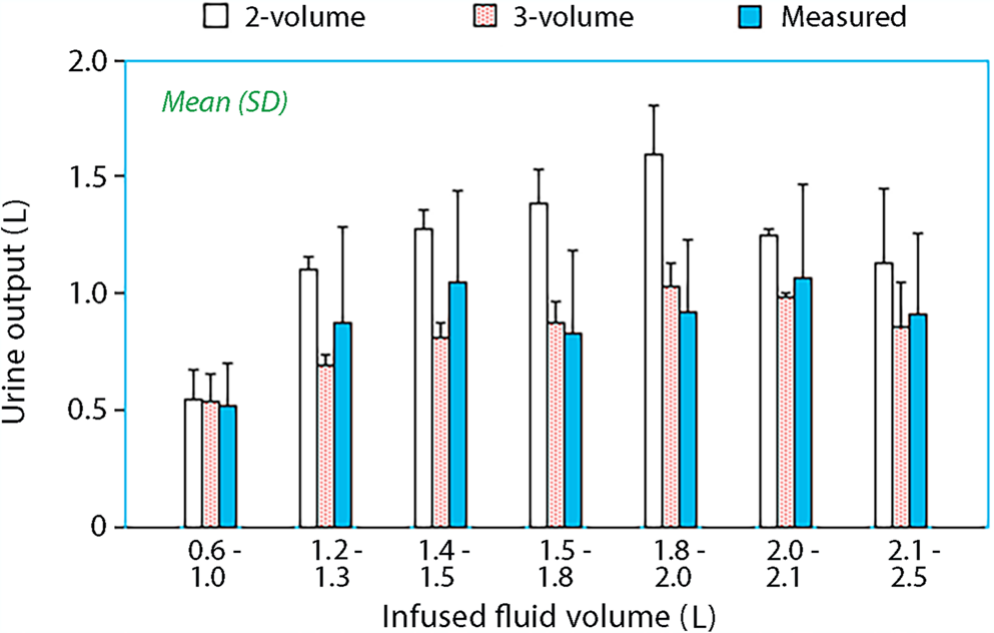
Simulated and measured urine output at the end of (180–240 min) infusion of various amounts of Ringer’s acetate in healthy volunteers. The two-volume model overestimates the urine output in ranges where the three-volume kinetic model should be preferred, starting at an infusion rate of 1.2 L. This graph is based on *post hoc* data that considered only the covariance effect of body weight on *V*_c_

Analysis of all experiments suggested that the two-volume model, but not the three-volume model, frequently overestimated the excreted urine volume, although the measured urine output served as an independent variable (Fig. S2). Dividing the cohort into 7 ranges of administered fluid suggested that the overestimation began following infusion of > 1.2 L of Ringer’s. Hence, the 2-volume model was not optimal for volumes larger than 1.2 L (Fig. 4**)**. The estimated sizes of *V*_c_, *V*_t1_, and *V*_t2_ for these fluid ranges are shown in Table 1. The original data are given in **Supplementary File 3.**

**Table 1:** The estimated volumes of *V*_c_, *V*_t1_, and *V*_t2_ for gradually increasing volumes of infused fluid, based on *post-hoc* data for four intervals of infused fluid (same ranges as in Fig. 4)

Infused volume (L)	Dilution end of infusion (mean ± SD)	V_c_ (L) (mean ± SD)	V_t1_ (median, IQR)	V_t2_ (median, IQR)	*N*
0.6–1.0	0.128 ± 0.042	3.8 ± 1.1	4.4 (3.2–8.9)	0	15
1.1–1.3	0.164 ± 0.046	3.8 ± 0.9	2.9 (1.4–3.4)	2.5 (2.2–8.2)	5
1.4–1.5	0.180 ± 0.044	3.5 ± 0.8	3.1 (2.0–5.9)	10.7 (5.2–115.8)	8
1.5–1.8	0.168 ± 0.049	3.7 ± 0.9	5.3 (4.2–6.8)	15.6 (3.2–34.5)	20
1.8–2.0	0.197 ± 0.066	4.0 ± 1.1	6.4 (5.1–7.9)	9.3 (4.4–27.6)	22
2.0–2.1	0.250 ± 0.067	4.4 ± 1.0	6.6 (5.5–7.9)	28.0 (10.2–83.8)	11
2.1–2.5	0.216 ± 0.073	5.1 ± 1.4	7.5 (6.0–10.3)	86.2 (18.3–208.0)	20

The size of *V*_c_ is a kinetic output parameter, *V*_t1_ = *V*_c_ *k*_12_/*k*_21_, and *V*_t2_ = *V*_t1_ *k*_23_/*k*_32_

The measured plasma dilution increased only mildly with increasing infusion volumes (Fig. S3, Table 1). Similarly, the measured urine output increased very slowly after infusion of 1.2 L of fluid (Fig. 4**)**.

### Study 3

The infused volume of Ringer’s was 1,897 ± 295 mL (lowest 1,195 mL) and plasma dilution was measured on 3,003 occasions. Urine was measured on 132 occasions. The three-volume model was statistically justified, given the Akaike criterion of − 11,151 (two-volume model − 11,060). The time periods 0–15, 15–20, 20–25, 25–30, 30–35, and 35–50 min were examined as covariates to *k*_23_, which governed the entrance of fluid to *V*_t2_. After 50 min, *k*_23_ was represented by the “typical value” (tv) for the group.

**Fig. 5:**
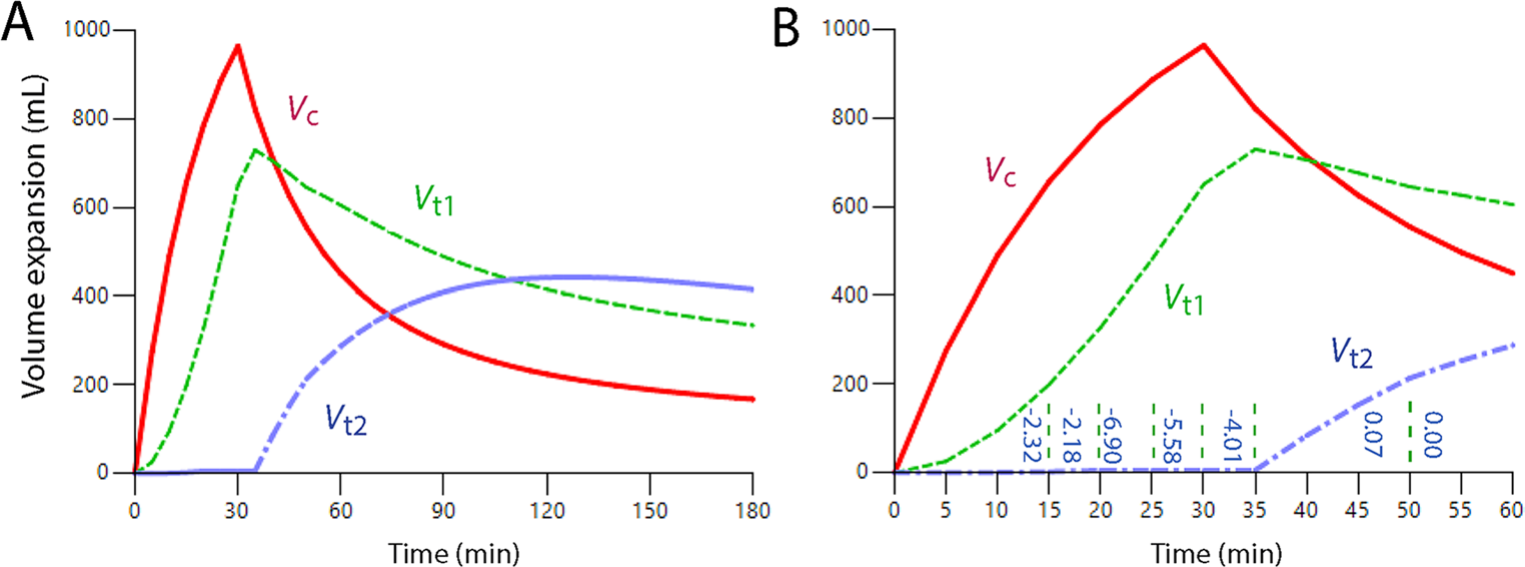
Simulated fluid distribution of an infusion of 1,897 mL of crystalloid fluid over 30 min in healthy volunteers **(A)** Simulation over 3 h **(B)** Close-up of the first hour only. Vertical lines indicate the intervals for which covariate analysis was applied to detect when *k*_23_ opens for the accumulation of infused fluid. The covariance value is given in numbers. Here, the covariance was applied from the start of the time interval, and the covariance factor is given in vertical text for each interval. The rate of flow from *V*_t1_ to *V*_t2_ at each point in time is given by: [(*v*_t1_ – *V*_t1_) * 0.0224 * (e ^ covariance factor)], where e = 2.718

Two kinetic analyses were made. The first was based on the covariate effect *at the end* of each time interval. The parameter estimates are shown in Table 2 and illustrated in Fig. 5. Here, the entrance of fluid into *V*_t2_ was strongly inhibited for up to 35 min of the experiment (i.e., 5 min after the infusion ended). Goodness-of-fit and performance measures are given in Fig. S4**.** The original data are given in **Supplementary File 4.**

**Table 2:** Population kinetic parameters for infused fluid volume in the final model. Shown are the typical values (tv) for the fixed parameters in the group, followed by individual-specific and time-specific covariates. The tv value for *k*_23_ is valid from 50 min and onward while *k*_23_ for the from the end of the preceding time periods was modified by covariates. The exponential covariate model was used in all instances except for the influence of the body weight on *V*_c_, which was a power model

Kinetic parameter	Covariate	Best estimate	95% CI	CV%	−2 LL
tv*V*_c_ (L)		4.62	4.36–4.88	2.9	
tv*k*_12_ (10^− 3^ min^− 1^)		36.5	33.2–39.7	4.6	
tv*k*_21_ (10^− 3^ min^− 1^)		23.7	21.1–26.4	5.7	
tv*k*_23_ (10^− 3^ min^− 1^)		22.4	19.0–25.8	7.7	
tv*k*_32_ (10^− 3^ min^− 1^)		17.0	15.2–18.9	5.5	
tv*k*_10_ (10^− 3^ min^− 1^)		14.2	12.7–15.8	5.4	−11,182
*V*_c_	Body weight	0.82	0.72–0.92	6.4	−11,192
*k*_10_	0.9% NaCl	−0.90	(−1.15)–(−0.64)	−14.2	−11,201
*k*_23_	0–15 min	−2.32	(−2.40)–(−2.25)	−1.5	
	15–20 min	−2.18	(−2.24)–(−2.12)	−1.3	
	20–25 min	−6.90	(−10.9)–(−2.89)	−29.6	
	25–30 min	−5.58	(−6.32)–(−4.85)	−6.7	
	30–35 min	−4.01	(−4.10)–(−3.91)	−1.2	
	35–50 min	0.072	0.069–0.074	1.6	−11,225
k _21_	During infusion	−4.87	(−5.12)–(−4.62)	−2.6	−11,236

tv = typical value for the group. CI = confidence interval. CV% = coefficient of variation (inter-individual).LL = log likelihood for the model during development. Mean body weight 76 kg,

The second kinetic analysis was based on the covariate effect *at the beginning* of the interval. The parameter estimates are shown and illustrated in Table S3 and Fig. S5 of **Supplementary File 1.** The entrance of fluid into *V*_t2_ was then strongly inhibited for up to 30 min, followed by slow filling of *V*_t2_ for up to 50 min and then a higher filling rate.

A comparison between the two analyses thus suggests that the *V*_t2_ opened between 30 and 35 min of the experiment.

## Discussion

### Key findings

This report consists of three parts, each highlighting a different aspect of fluid accumulation in the slow-exchange interstitial fluid space, *V*_t2_ (the “third space”). This compartment, *V*_t2_, after initially being virtually closed, begins to accumulate fluid at a specific point in time during an infusion of crystalloid fluid. The hemorrhage experiment data support P_if_ as a key element in explaining how much fluid is accumulated in *V*_t2_. The study of urine residuals suggests that *V*_t2_ opens after the infusion of approximately 1.2 L of Ringer’s over 30 min. The third sub-study supports that *V*_t2_ opens and begins to accumulate fluid at a specific point in time. Hence, the findings confirmed the study hypothesis.

### Interstitial pressure

The collective results of the present study agree with Guyton’s findings of a dramatic decrease in the resistance to volume expansion of the interstitium (x100,000) when its sub-atmospheric pressure passes zero and becomes positive [9–11]. Data from the dog suggest a quantitative relationship between interstitial volume expansion and P_if_. For example, Hopkinson et al. reported that P_if_ is −2.7 mmHg at baseline [21], and because an increase in *V*_t1_ of 2% is believed to increase P_if_ by 1 mmHg [26], then a 5% increase of *V*_t1_ would be required for P_if_ to reach zero in the normovolemic experiments in Study 1. The size of *V*_t1_ at baseline was 9.4 L (given by *V*_c_ *k*_12_/*k*_21_) and the fluid-induced increase in *V*_t1_ of 700 mL then represented a 7.4% greater *V*_t1_, which then caused *V*_t2_ to open (Fig. 2A). However, when bleeding occurs, the interstitial fluid translocates to the plasma by “capillary refill,” which decreases P_if_ [27]. Hopkinson et al.. further reported that normotensive bleeding in dogs decreased *P*_if_ by 3.9 mmHg, which, in the larger hemorrhage in the present study, would require expansion of *V*_t1_ by approximately 13% for P_if_ to pass zero [21]. The increase in *V*_t1_ of 1.1 L in the most extensive hemorrhage is 12% of the interstitial volume, which is close to the estimated “threshold,” and then allowed only very small amounts of fluid to enter *V*_t2_.

### How much fluid can be infused?

In the present study, the opening of *V*_t2,_ which is assumed to correspond to P_if_ > 0, occurred when *V*_t1_ had been expanded by 700 mL. This normally requires infusion of 1.2 L crystalloid fluid over 30 min in a conscious healthy adult. However, these values are not fixed but varies depending on to the physiological situation. For example, the infused volume needed before *V*_t2_ opens is higher if the fluid is administered at a slower rate because relatively more fluid then has time to be excreted during the pressure build-up in *V*_t1_ [7].

The infusion volume is also likely to be different if the pre-infusion P_if_ has been altered. An example is general anesthesia, where *V*_t1_ needs to be expanded by approximately 950 mL before *V*_t2_ opens [7]. This change is probably due to the redistribution of interstitial fluid to the plasma that occurs in response to anesthesia-induced decrease of the arterial pressure [27].

As suggested by Hopkinson et al., the infusion of hyper-oncotic colloid fluid and hemorrhage decrease P_if_ by means of promoting capillary refill, which pushes the threshold upward [21].

The threshold can also move downward – acute inflammation due to cholecystitis and appendicitis showed a threshold of slightly below 400 mL, corresponding to infusion of 870 mL; the same volume given to healthy volunteers did not open *V*_t2_ at all [7]. The low threshold in acute inflammation is probably due to an interstitial “suction pressure” created by cytokines and vasoactive molecules [29, 30].

Finally, the amount of fluid that can be infused before *V*_t2_ opens might be modified by aberrant filling of *V*_t1_, such as by disease-induced imbalance between capillary leakage and lymphatic flow. For example, the distribution of albumin between the plasma and interstitium can be disturbed by shedding of the endothelial glycocalyx layer [31]. Maldistribution of fluid is also promoted by inhibition of the lymphatic pumping due to cytokines, nitric oxide, anesthetic drugs and mechanical ventilation [32].

### ***The threshold for V***_t2_***opening is precise***

The third sub-study shows that fluid accumulation in *V*_t2_ is not a continuous process but is instead initiated at a specific point in time, in agreement with Guyton’s findings regarding a sudden decrease in the interstitial resistance for volume expansion. The analysis was based solely on data from healthy volunteers, which was a choice made to achieve mechanistic clarity by facilitating the identification of the threshold for the opening of *V*_t2_ in a reasonably well-defined physiological setting.

The evidence for the existence of a specific time-point for the opining of *V*_t2_ in the present cohort consists of the strongly negative time-specific covariances for *k*_23_, which prevented the entrance of fluid to *V*_t2_, as observed during several 5 min periods before the 30 min infusion of crystalloid fluid ended. Slightly different outputs were obtained when the covariance was taken at the end or at the beginning of the time periods. In the latter analysis, entrance of fluid to *V*_t2_ had been initiated at the end of the 30–35 min interval (Fig. S1), while the other plot confirmed that no fluid had entered *V*_t2_ in the beginning of that interval (Fig. 4).

### Changes with increased infusion volumes

The 7 ranges of gradually increasing infusion volumes showed that the urine output increased very slowly after 1.2 L of fluid had been infused (Fig. 3**)**, which is consistent with the only slight increases observed in renal clearance of fluid in overhydration settings [33]. The plasma dilution also increased slowly when more fluid was administered, but the relationship [ΔDilution/ΔInfused volume] was similar during infusion (when *V*_t2_ was closed) and after the infusion (when *V*_t2_ was open) (Fig. S3).

The sizes of the expandable fluid compartments adapted depending on the amount of fluid administered (Table 1**)**. The central volume, *V*_c_, increased only with the largest infused volumes, as evidence of “stress-relaxation.” This implies that a rapid development of hypervolemia increases the ability of the circulation to hold an expanded blood volume [34]. “Stress-relaxation” decreases the measured plasma dilution but not the calculated plasma volume expansion.

The initial size of *V*_t1_ was similar to the size of *V*_c_, but a gradual increase occurred when more than 1.5 L was infused.

The size of *V*_t2_ varied greatly, even attaining supraphysiological values. The sum of *V*_t1_ and *V*_t2_ is assumed to correspond roughly to the expandable parts of the physiological interstitial space, which is normally smaller than the 18–20% of the body weight measured using small tracer molecules [35]. The very large volumes of *V*_t2_ suggest an absence of free flow and the possible formation of lacunae of free fluid, as has been demonstrated in overhydrated animals [17–19].

The turnover of fluid in *V*_t2_ is the slowest of the three compartments (Fig. 4A) and supports the assumption that *V*_t2_ corresponds to the interstitial gel phase, where restricted fluid movement is known to occur. The fast-exchange compartment, *V*_t1_, probably corresponds to the free fluid phase in the interstitium and lymphatic vessels.

### Methodological issues

The amount of crystalloid fluid that can be infused before *V*_t2_ opens is difficult to study because a larger group of experiments is needed to obtain reasonably precise estimates of the parameters in the 3-volume kinetic model. Moreover, the requirement for “good” data is higher when the compartments are connected in a serial chain rather than as a parallel setup [28].

There is sometimes a problem that a mixed-model analysis forces all experiments into a single model. Covariates can be used to account for parameter changes occurring gradually when more fluid is infused, but this is not always successful. Therefore, in the second sub-study, the analysis was performed in four ranges of infused fluid, and *post hoc* data were used to further break down the output into narrower ranges. An approach was introduced whereby the modeled urinary excretion was compared to the measured urine output. The 2-volume model then overestimated the excreted urine when more than 1.2 L was infused, suggesting model imperfection. The conventional way to decide which model is statistically justified is to apply the *F* test [22] or the Akaike information criterion [25], but they require that the 3-volume model can be applied. The urine residual approach used here needs only the measured urine output and a reasonably accurate kinetic analysis according to the two-volume model, which is far less demanding than an analysis according to the 3-volume model. However, the urine residual approach is not suggested for statistical purposes, but only as a screening tool to indicate when *V*_t2_ opens and begins to accumulate fluid.

### Kinetic model

Volume kinetics is an adaptation of pharmacokinetic theory applicable to infusion fluids. It differs from drug kinetics by assuming that the compartment walls are expandable. In addition, plasma dilution based on blood hemoglobin is used as a replacement for fluid concentration, as this cannot be measured due to the already high concentration of water in the blood (80%) [20]. The mathematical handling of the data is the same as in conventional drug pharmacokinetics.

Volume kinetics is a whole-body method and provides a summary measure of the fluid distribution in the body. Likewise, the reported covariate effects pertain to their total effect on the studied volunteer. Nevertheless, the P_if_ and the interstitial compliance for volume expansion is known to vary greatly among tissues. In-depth reviews of these local variations have been given by Aukland, Reed, and Wiig [36, 37].

The mixed models method applied to the data is an industrial standard approach. It estimates kinetic parameters with good precision in a series of experiments in which individual and time-dependent differences are disclosed by covariate analysis [24]. The mixed models approach was particularly useful in the third sub-study, as the covariance analysis could mathematically separate 6 short time periods from the last parts of the experiments regarding the parameter *k*_23_. Technically, this was done by marking a column in the spreadsheet with numbers derived by giving each point in time a digit from 0 to 6. This column was handled as a single covariate with 7 levels. Only the confidence interval served as a guide for the appropriateness of each level because only one reduction of the likelihood for the model was obtained for all 7 levels. With the optimal curve-fit, the addition of these 6 covariates to the model decreased its likelihood by 24 steps, whereas a single parameter requires 3.8 steps to achieve a statistical significance of *P* < 0.05.

### Clinical implications

The *V*_t2_ space probably serves mainly as a reservoir for excess fluid to prevent overloading of *V*_t1_ and, indirectly, the circulation. This preventive effect is due to the long turnover time for fluid in *V*_t2_, which effectively shuts off the pool from the central circulation. Another consequence of the long turnover time is that the half-life of infused crystalloid fluid is prolonged by several multiples, which explains why the body weight can remain increased several days after surgery [7]. However, the opening of *V*_t2_ might also be related to the disruption of the cytoarchitecture and the pathological changes of the heart muscle observed after overhydration in animals [17–19].

The clinical importance of “third-spacing” in humans remains unclear because *V*_t2_ has not been included in studies of overhydration. However, there is still reason to suspect that “third-spacing” is part of the explanation as to why overhydration is associated with increased complications and higher mortality in surgical and intensive care patients [12–16].

The type of “third-spacing” that occurs in severe inflammatory disorders (like sepsis) is more troublesome to the circulation because the accumulation of fluid in *V*_t2_ competes more strongly with the lymphatic flow than “third-spacing” due to overload [38, 39]. The maldistribution of fluid and albumin that develops includes the triad hypovolemia, hypoalbuminemia, and peripheral edema [8].

### Limitations

The experimental data used in this report were taken from a database of collected experiments performed during the past 25 years. Only healthy volunteers who received crystalloid fluid over 30 min were studied. Very consistent protocols have been used during the development of volume kinetics, which was initiated in the early 1990s.

The infusion rates used are higher than usually practiced, but they occur. Notably, more fluid accumulates in *V*_t1_ over time if an infusion is continued and the diuretic response to the plasma volume expansion is poor, such as is the case during general anesthesia [7, 8].

Infusion therapy may also consist of colloid fluid. The effect of nearly iso-oncotic colloid fluid (5% albumin, Voluven, and Gelofusine) on the opening of *V*_t2_ must be slight because the capillary leakage rate of the infused volume is very slow. In a recent study, the capillary leakage rate of Gelofusine during surgery was only 1/20 of the rate reported for crystalloid in the present study [39]. Hyper-oncotic colloids, such as 20% albumin, are even likely to decrease P_if_ by translocating interstitial fluid to the plasma [40]. A reduction of P_if_ from infusion of hyper-oncotic dextran has been demonstrated in the dog [21].

The focus of the present kinetic analysis was on the opening of *V*_t2_, so this left little space for the inclusion of other potential covariates, as the program used cannot handle more than 15 parameters at one time. Other studies have shown that the arterial pressure has little influence over fluid distribution in healthy volunteers, whereas the composition of the blood (e.g., high red cell and platelet counts) slightly increases the rate of fluid distribution via *k*_12_ [32]. Another issue regarding the modeling is that the covariates were added in blocks (e.g., levels of blood loss) (Study 1) and time intervals (Study 3), meaning that the entire block had to be evaluated for statistical justification. The validity of each covariate had to be judged based on the associated confidence interval.

Not all covariates that were significant in the analysis of Study 3 were found in Study 1, and the reason is likely to be the lower power of Study 1. First, the size of *V*_c_ increases with body weight, as has been shown in previous analyses [6, 7, 28]. Here, the quite small spread of the data might have contributed to the lack of significance. Second, *k*_21_ is usually inhibited during a fast infusion of fluid [28]. This is believed to be due to the temporarily increased venous pressure during volume loading, which limits the return flow of the distributed fluid to the plasma (mainly via the lymphatic flow). This covariate was close to attaining significance in Study 1, but it did not reach the preset limit for inclusion.

Ten patients who received isotonic saline were included in Study 3. Previous analyses show that this fluid retards the urine output while otherwise having the same parameter values as observed with Ringer’s infusion [41]. Here, the deviation from the kinetics of Ringer´s solution was adjusted by covariance analysis.

## Conclusions

Three sub-studies explored the prerequisites for the accumulation of fluid in the slow-exchange interstitial fluid space (“third fluid space,” *V*_t2_). The first study pointed out that the interstitial fluid pressure, P_if_, is an important driving force for this accumulation. The second study suggests that infusion of > 1.2 L of crystalloid fluid is required to initiate fluid accumulation in *V*_t2_. The third study showed that fluid begins to enter *V*_t2_ at a specific point in time during an infusion, whereas the space is virtually closed for volume expansion prior to that event. These findings confirm and add to existing knowledge and are relevant to anesthesiology and intensive care practice.

## Supplementary Information

Below is the link to the electronic supplementary material.

Supplementary Material 1 (DOCX 15.9 MB)

Supplementary Material 2 (XLSX 112 KB)

Supplementary Material 3 (XLSX 396 KB)

Supplementary Material 4 (XLSX 432 KB)

## Data Availability

All data supporting the findings of this study are available within the paper and its Supplementary Information (Supplementary Files 2, 3, and 4).
