# Supplementary material for: When does the “third fluid space” open?
Source: Pflugers Arch. 2025 Dec 17;478(1):12. doi: 10.1007/s00424-025-03135-y (PMC12708698; doi:10.1007/s00424-025-03135-y)
Supplement: Supplementary file 1 — Supplementary Material 1 (DOCX 15.9 MB) [file 424_2025_3135_MOESM1_ESM.docx]

**Supplementary File**

When does the “third fluid space” open?

**__________________________________________________________________________**

**Content**

1. Manuscript where the data were first used.

Table S1 – ethics approvals.

1. Fig. S1. Performance measures for Study 1.
2. Table S2. Kinetic parameters for Study 1.
3. Fig. S2 and S3. Urine residuals and plasma dilution in in Study 2.
4. Fig. S3. Performance measures for Study 3.
5. Table S3. Kinetic parameters for Study 3, second run.
6. Fig. S4. Opening of *V*_t2_, second run.
7. Phoenix program file.

**__________________________________________________________________________**

**Study 1** used data from the following paper:

Drobin D, Hahn RG. Volume kinetics of Ringer’s solution in hypovolemic volunteers. Anesthesiology 1999:90:81–91.

**Study 2** also used data from the following papers:

Hahn RG, Drobin D, Ståhle L. Volume kinetics of Ringer’s solution in female volunteers. Br J Anaesth 1997: 78: 144–148.

Drobin D, Hahn RG. Kinetics of isotonic and hypertonic plasma volume expanders. Anesthesiology 2002: 96: 1371–1380.

Svensén C, Drobin D, Olsson, J, Hahn RG. Stability of the interstitial matrix after crystalloid fluid loading studied by volume kinetic analysis. Br J Anaesth 1999: 82: 496–502.

Hahn RG, Lindahl C, Drobin D. Volume kinetics of acetated Ringer’s solution during experimental spinal anesthesia. Acta Anaesthesiol Scand 2011; 55: 987–994.

Hahn RG, Bergek C, Gebäck T, Zdolsek J. Interactions between the volume effects of hydroxyethyl starch 130/0.4 and Ringer’s acetate. Crit Care 2013; 17: R104.

Zdolsek J, Li Y, Hahn RG. Detection of dehydration by using volume kinetics. Anesth Analg 2012; 115; 814–822.

**Study 3** excluded data from the (Zdolsek et al) and added data from Anesthesiology 2002 and unpublished data.

**Table S1.** The manuscripts that provided data for the analysis, with ethics approval numbers.

| Males /  females | Infusions  used | Publication | Ethics approval | Committee |
| --- | --- | --- | --- | --- |
|  |  |  |  |  |
| 10 / 0 | **30^1^** | Anesthesiology 1999, 90, 81-91 | 54/95 | Huddinge Hospital |
| 10 / 0 | **13** | Br J Anaesth 1999, 82, 496-502 | 54/95 | Huddinge Hospital |
| 0 / 6 | **6** | Br J Anaesth 1997: 78: 144-8 | 168/91 | Huddinge Hospital |
| 10 / 0 | **30** | Anesthesiology 2002, 96, 1371-80 | 228/98 | Huddinge Hospital |
| 10 / 0 | **17** | Anesth Analg 2012; 115; 814-22 | M114-09 | Linköping |
| 0 / 9 | **9** | Acta Anaesthesiol Scand 2011, 55, 987-94 | 123/97 | Huddinge Hospital |
| 10 / 0 | **10** | Crit Care 2013, 17, R104 | 2009/1091-31/2 | Stockholm |
| 20 / 0 | **20** | No separate publication | 115–00 + 168/91  2007/851-31/4 | Huddinge Hospital Stockholm |
|  |  | All Committees are located in Sweden |  |  |

^1^The 20 experiments that included hemorrhage were only used in Study 1.

**
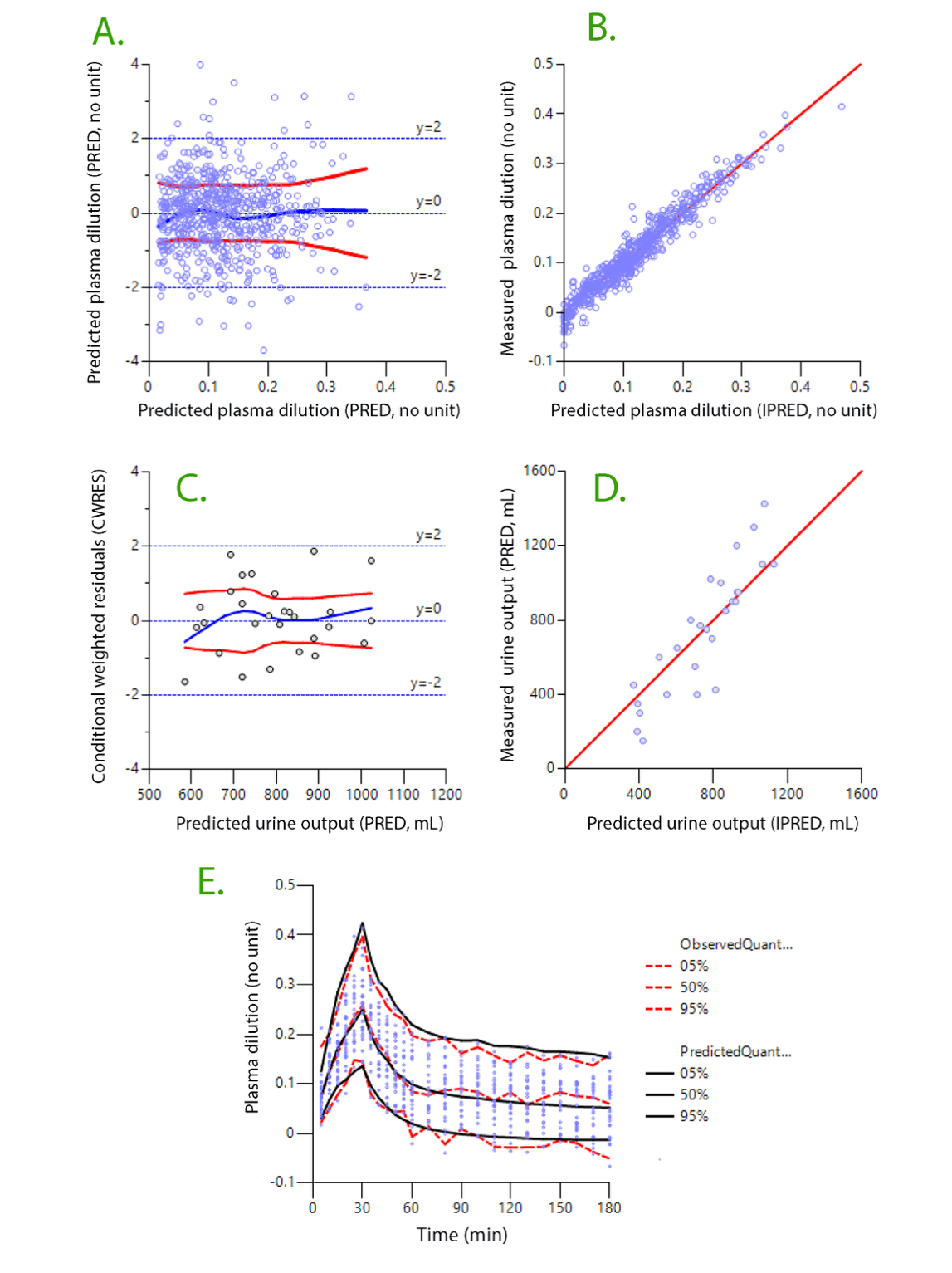
**

**Fig. S1. Performance measures for the hemorrhage experiments (Study 1)**

**A:** The conditional weighted residuals (CWRES) *versus* the predicted plasma dilution (without covariates) for all experiments, including those that involved hemorrhage. Blue line shows the LOESS (logically weighted scatterplot smoothing) line for the residuals, red top line a LOESS fit to the absolute residuals, and the irregular blue lines show y = ± 2 SD for the residuals.

**B:** Measured plasma dilution *versus* the predicted dilution (with covariates).

**C:** The conditional weighted residuals (CWRES) *versus* the predicted urine output for all experiments (without covariates), including those that involved hemorrhage.

**D:** Measured urine output *versus* the predicted urine output (with covariates).

**E:** Predictive check. The measured plasma dilution and its 95% confidence interval (“Observed”) is compared to the confidence interval for 1,000 simulations (“Predicted”) based in the optimal model parameters. Plots are outputs from the Phoenix program.

**Table S2. Kinetic parameters for Study 1.**

Population kinetic parameters for infused fluid volume in the final model.

Shown are the typical values (tv) for the fixed parameters in the group, followed by covariates relation to the hemorrhage group. The exponential covariate model was used throughout.

| Kinetic parameter | Covariate | Best estimate | 95% CI | CV% | -2 LL |
| --- | --- | --- | --- | --- | --- |
|  |  |  |  |  |  |
| tv*V*_c_ (L) |  | 4.05 | 3.68–4.43 | 4.7 |  |
| tv*k*_12_ (10^-3^ min^-1^) |  | 50.5 | 42.3–58.7 | 8.3 |  |
| tv*k*_21_ (10^-3^ min^-1^) |  | 21.8 | 18.1–25.4 | 8.5 |  |
| tv*k*_23_ (10^-3^ min^-1^) |  | 33.9 | 27.9–40.0 | 9.1 |  |
| tv*k*_32_ (10^-3^ min^-1^) |  | 30.1 | 26.2–33.9 | 6.5 |  |
| tv*k*_10_ (10^-3^ min^-1^) |  | 16.9 | 12.8–21.1 | 12.5 | -2389 |
| *k*_23_ | During infusion | -6.79 | (-7.38)–(-6.20) | -4.4 | -2393 |
|  | 450 mL bleed | -0.22 | (-0.25)–(-0.19) | -6.8 |  |
|  | 900 mL bleed | -2.90 | (-3.59)–(-2.20) | -12.2 | -2401 |
| *k*_10_ | 450 mL bleed | -0.28 | (-0.37)–(-0.20) | -15.0 |  |
|  | 900 mL bleed | -0.83 | (-1.08)–(-0.59) | -15.0 | -2407 |
| *V*_c_ | 450 mL bleed | -0.06 | (-0.07)–(-0.15) | -9.8 |  |
|  | 900 mL bleed | -0.25 | (-0.34)–(-0.19) | -19.6 | -2412 |
|  |  |  |  |  |  |

tv = typical value for the group. CI = confidence interval. CV% = coefficient of variation (inter-individual).

LL = log likelihood for the model during development. Mean body weight 78 kg,

**
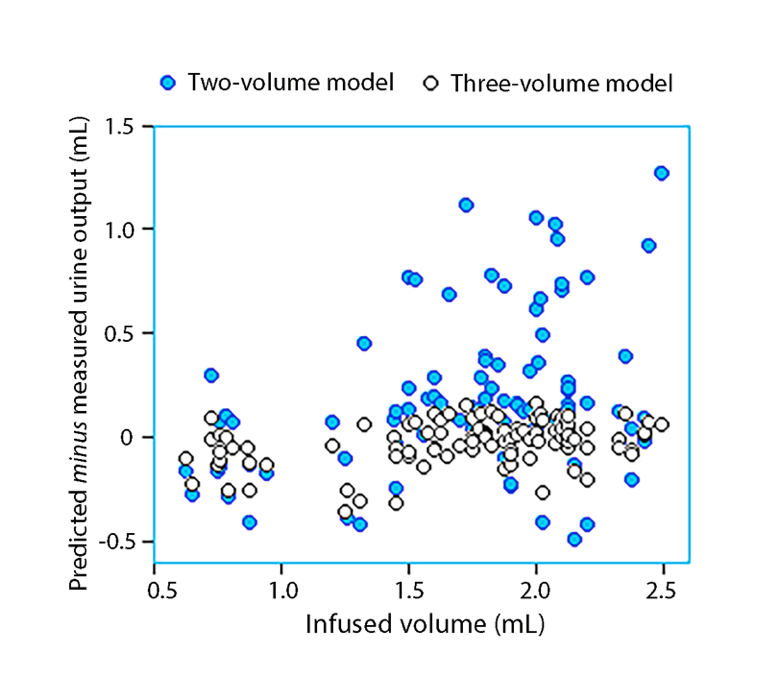
**

**Fig. S2. Urine residuals.**

Population analysis was performed using both the two-volume and three-volume model (including all covariates) after which the infused volume was plotted *versus* the difference model-predicted and measured urine output.


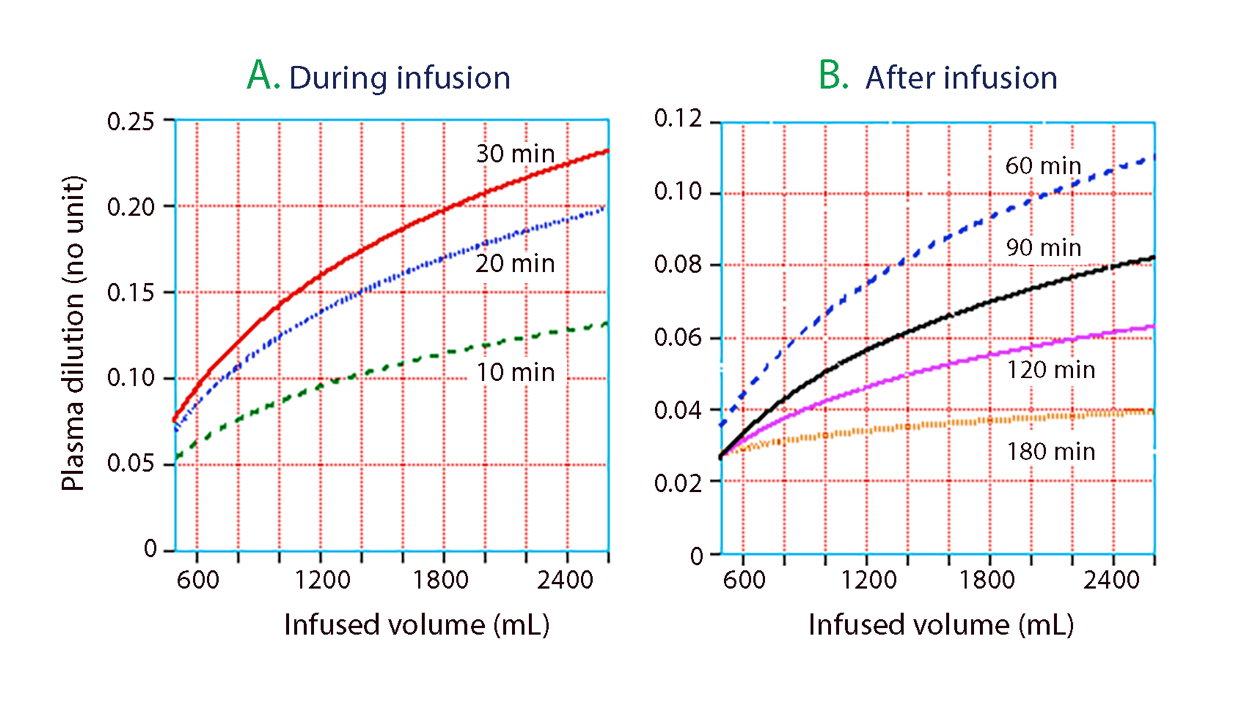


**Fig. S3. Plasma dilution.**

Linear regression analysis of measured plasma dilution, which is an index of plasma volume expansion, for increasing volumes of infused Ringer´s solution. Isobars show the plasma dilution as a specific point in time. All infusions lasted 30 min (N=101). Logarithmic conversion provided the best curve-fit.


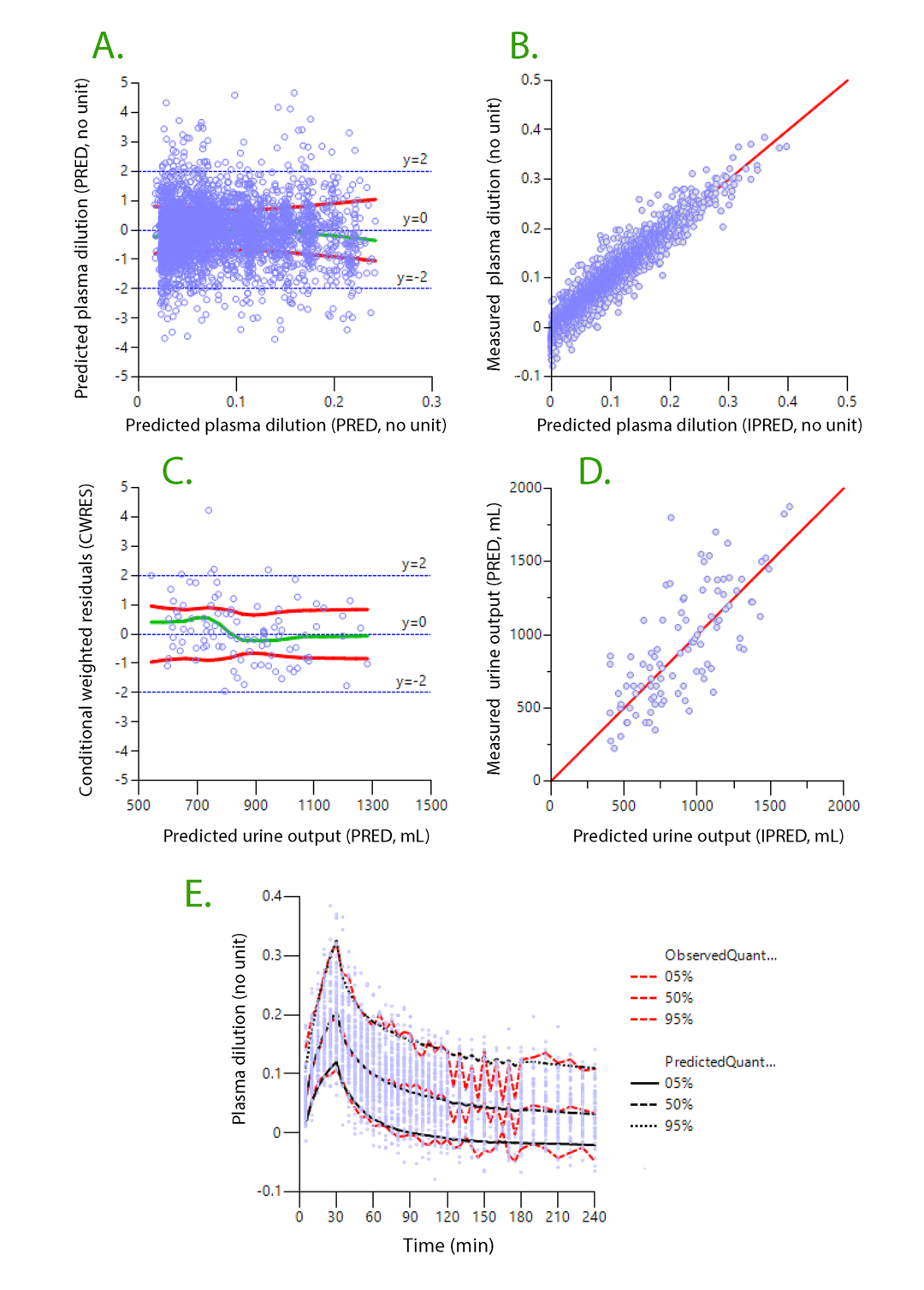


**Fig. S4. Performance measures for the experiments in Study 3**

**A:** The conditional weighted residuals (CWRES) *versus* the predicted plasma dilution (without covariates) for all experiments, except those that involved hemorrhage.

**B:** Measured plasma dilution *versus* the predicted dilution (with covariates).

**C:** The conditional weighted residuals (CWRES) *versus* the predicted urine output for all experiments (without covariates), including those that involved hemorrhage. Good model specification is indicated by the random distribution of the data around the individual predictions, and few data points > ± 3 SD.

**D:** Measured urine output *versus* the predicted urine output (with covariates).

**E:** Predictive check. The measured plasma dilution and its 95% confidence interval (“Observed”) is compared to the confidence interval for 1,000 simulations (“Predicted”) based in the optimal model parameters. Plots are outputs from the Phoenix program.

**Table S3**

Population kinetic parameters for infused fluid volume in the final full block model.

Shown are the typical values (tv) for the fixed parameters in the group, followed by individual-specific covariates and time-specific. The tv value for *k*_23_ is valid from

50 min and onward while *k*_23_ for the preceding time periods was modified by covariates.

This analysis was made with **forward direction**, which means that reported value pertains to the beginning of the studied range.

| Kinetic parameter | Covariate | Covariate model | Best estimate | 95% CI | CV% |
| --- | --- | --- | --- | --- | --- |
|  |  |  |  |  |  |
| ­­tv*V*_c_ (L) |  |  | 4.06 | 3.86–4.27 | 2.6 |
| tv*k*_12_ (10^-3^ min^-1^) |  |  | 65.7 | 62.2–69.3 | 2.8 |
| tv*k*_21_ (10^-3^ min^-1^) |  |  | 41.4 | 39.3–43.5 | 2.6 |
| tv*k*_23_ (10^-3^ min^-1^) |  |  | 18.8 | 16.5–21.1 | 6.2 |
| tv*k*_32_ (10^-3^ min^-1^) |  |  | 3.86 | 3.62–4.10 | 3.2 |
| tv*k*_10_ (10^-3^ min^-1^) |  |  | 15.4 | 14.6–16.2 | 2.7 |
| *V*_c_ | Body weight | Power | 0.79 | 0.77–0.82 | 1.7 |
| *k*_10_ | 0.9% NaCl | Exponential | -0.67 | (-0.82)–(-0.52) | -11.4 |
| *k*_23_ | 0-15 min | Exponential | -4.29 | (-4.53)–(-4.05) | -2.9 |
|  | 15-20 min | Exponential | -5.03 | (-5.69)–(-4.37) | -6.7 |
|  | 20-25 min | Exponential | -6.85 | (-15.1)–1.41 | -61.5 |
|  | 25-30 min | Exponential | -5.59 | (-6.03)–(-5.15) | -4.0 |
|  | 30-35 min | Exponential | -1.40 | (-1.50)–(-1.31) | -3.5 |
|  | 35-50 min | Exponential | 0.47 | 0.44–0.49 | 2.4 |
| *k*_21_ | During infusion | Exponential | -0.42 | (-0.48)–(-0.36) | -7.4 |
|  |  |  |  |  |  |

tv = typical value for the group. CI = confidence interval. CV% = coefficient of variation (inter-individual).

LL = log likelihood for the model during development. Mean body weight 76 kg,


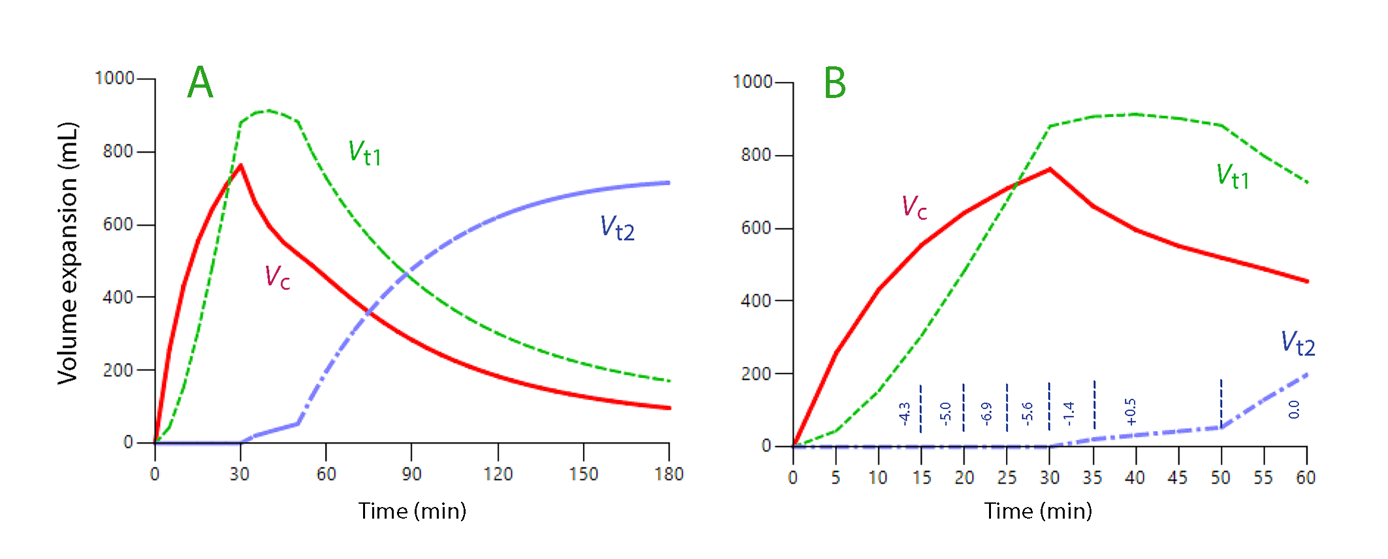


**Fig. S5. Opening of *V*_t2_**

Simulated distribution of an infusion of 1,897 mL of crystalloid fluid over 30 min in healthy volunteers.

**(A)** Simulation over 3 hours.

**(B)** Close-up of the first hour only. The intervals for which covariate analysis was applied to detect when *k*_23_ opens for accumulation of infused fluid are indicated by vertical lines. Here, the covariance effect was taken from the end of the time interval. The covariance factor is also given in vertical text for each interval.

**Phoenix program file**

Phoenix Program file used for simulation in of the data in Table S2. The differential equations for the kinetic model are highlighted by red color. Ke = *k*_10_. The “sleep” lines instruct the program to change parameter value after the time shown in parenthesis. Set tvk_23_ and tvk_32_ to zero is a two-volume model is desired.

test(){

deriv(A1 = - (A1 * Ke)- (A1 * K12- A2 * K21))

urinecpt(A0 = (A1 * Ke))

deriv(A2 = (A1 * K12- A2 * K21)- (A2 * k23- A3 * k32))

deriv(A3 = (A2 * k23- A3 * k32))

double(tvK21)

sequence{

tvK21=0.010

sleep(30)

tvK21=0.04139

}

double(tvk23)

sequence{

tvk23=0.0002564

sleep(15)

tvk23=0.000012234

sleep(5)

tvk23=0.00001983

sleep(5)

tvk23=0.0000699

sleep(5)

tvk23=0.00461

sleep(5)

tvk23=0.00257

sleep(15)

tvk23=0.0187

}

C = A1 / V

dosepoint(A1, idosevar = A1Dose, infdosevar = A1InfDose, infratevar = A1InfRate)

error(CEps = 0.0268931440925238)

observe(CObs = C + CEps)

error(A0Eps = 96.5731390598463)

observe(A0Obs = A0 + A0Eps)

C3 = A3 / V3

stparm(V = tvV * exp(nV))

stparm(Ke = tvKe * exp(nKe))

stparm(K12 = tvK12 * exp(nK12))

stparm(K21 = tvK21 * exp(nK21))

stparm(V3 = tvV3 * exp(nV3))

stparm(k23 = tvk23 * exp(nk23))

stparm(k32 = tvk32 * exp(nk32))

fixef(tvV = c(, 4456, ))

fixef(tvKe = c(, 0.0154, ))

fixef(tvK12 = c(, 0.0657, ))

fixef(tvV3 = c(, 10000, ))

fixef(tvk32 = c(, 0.00386, ))

ranef(diag(nV, nKe, nK21, nK12, nV3, nk23, nk32) = c(0.30355015, 1.4954665, 0.173917, 0.2311694, 2.2561062, 0.38804985, 3.5020762))
